# Fluorescent AIE-Active Materials for Two-Photon Bioimaging Applications

**DOI:** 10.3389/fchem.2020.617463

**Published:** 2020-12-14

**Authors:** Qing Lu, Cheng-Juan Wu, Zhiqiang Liu, Guangle Niu, Xiaoqiang Yu

**Affiliations:** ^1^State Key Laboratory of Crystal Materials, and Advanced Medical Research Institute, Shandong University, Jinan, China; ^2^College of Chemistry, Chemical Engineering and Material Science, Shandong Normal University, Jinan, China

**Keywords:** two-photon, aggregation-induced emission, fluorescent material, fluorescent detection, fluorescence imaging

## Abstract

Fluorescence imaging has been widely used as a powerful tool for *in situ* and real-time visualization of important analytes and biological events in live samples with remarkably high selectivity, sensitivity, and spatial resolution. Compared with one-photon fluorescence imaging, two-photon fluorescence imaging exhibits predominant advantages of minimal photodamage to samples, deep tissue penetration, and outstanding resolution. Recently, the aggregation-induced emission (AIE) materials have become a preferred choice in two-photon fluorescence biological imaging because of its unique bright fluorescence in solid and aggregate states and strong resistance to photobleaching. In this review, we will exclusively summarize the applications of AIE-active materials in two-photon fluorescence imaging with some representative examples from four aspects: fluorescence detection, *in vitro* cell imaging, *ex vivo* tissue imaging, and *in vivo* vascular imaging. In addition, the current challenges and future development directions of AIE-active materials for two-photon bioimaging are briefly discussed.

## Introduction

In recent years, fluorescence imaging has been widely used as a powerful tool for *in situ* and real-time visualization of important analytes and biological events in live samples with remarkably high selectivity, sensitivity, and spatial resolution (Ding et al., 2018; Zheng et al., 2019; Zhou et al., 2019; Liu et al., 2020; Lv Z. et al., 2020). Most of the structures and components in live samples do not emit fluorescence. Only when the fluorescent material specifically binds to the target, can the target be observed by the fluorescence microscope. Then the imaging and observation of ions, small biological molecules such as amino acids, sugars and cholesterol, biological macromolecules, various organelles, and the intracellular microenvironment at the cellular level are realized. Therefore, the development of fluorescence imaging depends not only on the microscopic technology, but also on the fluorescent materials.

**Scheme 1:**
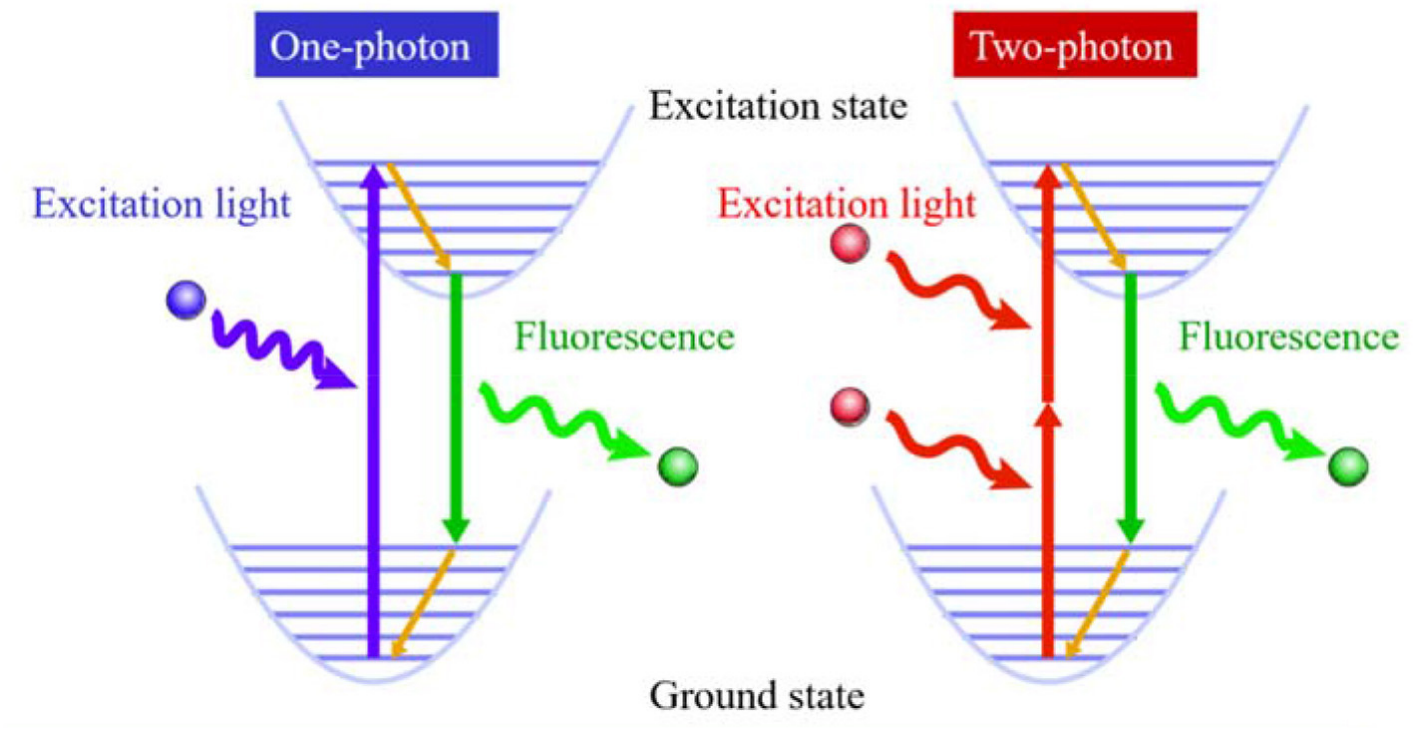
Principle of two-photon excited fluorescence for bioimaging.

Two-photon fluorescence bioimaging has incomparable advantages over one-photon (Scheme 1): first, it uses near-infrared (NIR) photon as the excitation source and causes mild photondamage to live samples (Looney et al., 2011); second, due to low excitation light scattering and NIR excitation, it can be used for deep penetrating imaging of tissues (Tadayon et al., 2019). Moreover, it emits light only when it is in the focus position in bioimaging, resulting in ultrahigh resolution (Song et al., 2020). Therefore, compared with one-photon fluorescence imaging, it is of great significance to perform two-photon fluorescence imaging to visualize important substances and biological processes in biological systems and reveal the mysteries of life systems (Gao Y.T. et al., 2020; Li Y. et al., 2020; Li Y.Y. et al., 2020; Zheng et al., 2020).

**Figure 1:**
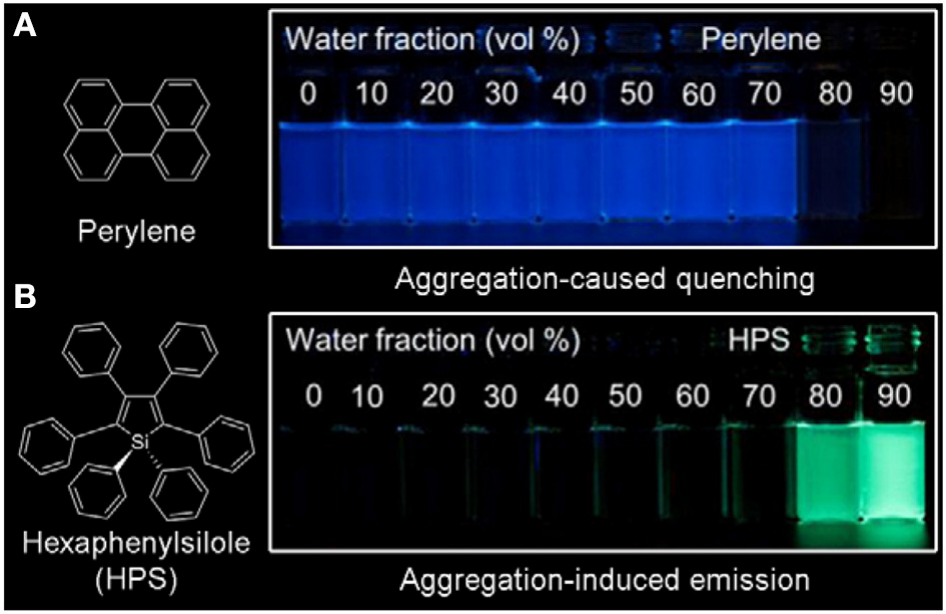
Fluorescent photographs of **(A)** perylene and **(B)** hexaphenylsilole (HPS) in THF/H_2_O mixture containing different water fractions under 365 nm UV irradiation (reproduced with permission from Mei et al., 2015; Copyright 2015 American Chemical Society).

However, the laser energy used in two-photon fluorescence imaging is relatively strong, and the photobleaching resistance of most traditional organic fluorescent materials is limited. One method to enhance their anti-photobleaching ability is increasing the concentration of traditional organic fluorescent materials, but they are prone to aggregation at high concentration in biological systems, which causes fluorescence quenching due to strong π-π stacking (Figure 1A). Another effective method is designing organic fluorescent materials with a large two-photon absorption cross section by introduction of strong electron donor and acceptor groups as well as extending the length of π-conjugation. As these strategies cause high hydrophobicity, the same problem of fluorescence quenching still exists. Thus, it is of great significance to develop new fluorescent materials to surmount the quenching problem of traditional hydrophobic fluorescent materials at a high concentration in a biological environment.

A new class of fluorescent materials (Figure 1B) with aggregation-induced emission (AIE) discovered by Tang's group and others have received considerable attention due to their unique photophysical properties (Luo et al., 2001; An et al., 2002, 2012; Shimizu et al., 2009; Shustova et al., 2013; Yan et al., 2015; Sasaki et al., 2016; Li and Li, 2017; Tsujimoto et al., 2017; Zhang et al., 2017; Ren et al., 2019; Guo et al., 2020; Hu et al., 2020; Kong et al., 2020; Mao et al., 2020; Qin et al., 2020). The widely accepted working mechanism of AIE-active fluorescent materials is restriction of intramolecular motions (Mei et al., 2015; Chen et al., 2019; Tu et al., 2020), thus the AIE materials generally show strong emission in aggregate and solid states and strong photobleaching resistance (Hong et al., 2011; Chen Y. et al., 2018; Cao et al., 2019; Li et al., 2019; Chen et al., 2020; Feng H. et al., 2020; He et al., 2020b; Huang et al., 2020; Li Q. et al., 2020; Ni et al., 2020; Wei et al., 2020; Xu Y. et al., 2020; Yin et al., 2020). On the other hand, AIE materials mostly exist in the form of nanoaggregates in biological systems, which are not easily discharged by the biological system through metabolism, enabling long-term dynamic tracking (Xie et al., 2019; Niu et al., 2020).

In a word, AIE materials are an ideal choice for two-photon fluorescence imaging. Recently, many AIE materials have been rationally designed and developed for two-photon fluorescence bioimaging (Gao et al., 2015; Wang et al., 2015, 2019a; Xiang et al., 2015; Zhu et al., 2015; Jiang et al., 2017; Yang et al., 2017; Chen M. et al., 2018; Yan et al., 2018; Gao Y. et al., 2020; Zhang R. et al., 2020). However, reviews on the application of AIE-active fluorescent materials for two-photon fluorescent bioimaging are rarely reported, except one review article about two-photon organic AIE dots (Lou et al., 2016). Considering some reviews have summarized the applications of AIE materials for cancer therapy (Gao and Tang, 2017; Hu et al., 2018; Zhang et al., 2018; Dai et al., 2020; He et al., 2020a; Wu and Li, 2020), in this review, we will focus on the AIE-active materials for two-photon bioimaging. This review summarizes the latest literatures in the past 6 years and discusses the two-photon bioimaging applications of AIE-active materials from four aspects: fluorescence detection, *in vitro* cell imaging, *ex vivo* tissue imaging, and *in vivo* vascular imaging. At the end of this review, the future directions and developments of AIE-active materials for two-photon bioimaging will be discussed.

## Bioimaging Applications

### Fluorescent Detection

**Figure 2:**
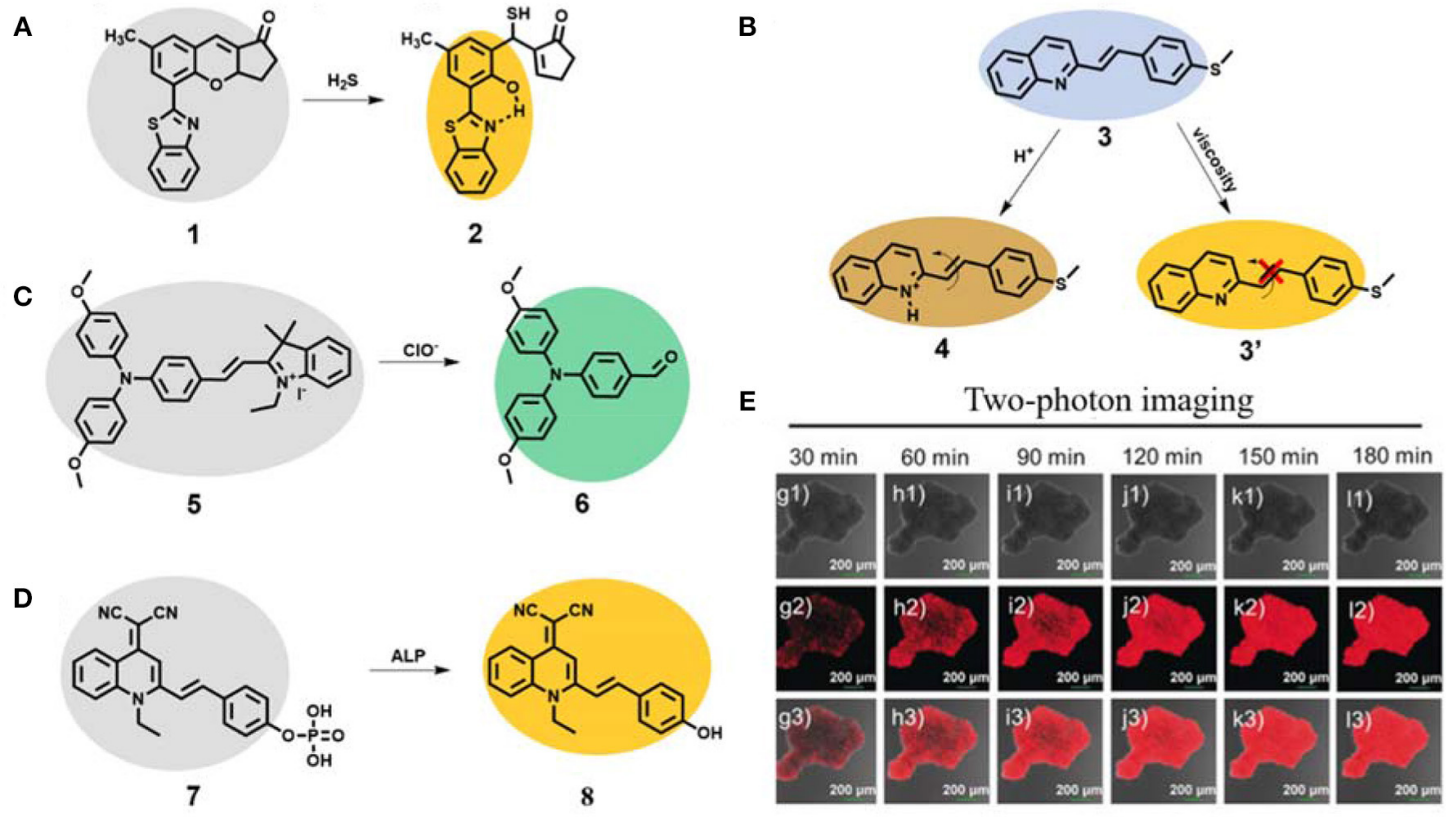
**(A)** Working principle of AIE probe **1** for fluorescent two-photon detection of H_2_S. **(B)** Working principle of AIE probe **3** for fluorescent two-photon detection of pH and viscosity. **(C)** Working principle of AIE probe **5** for fluorescent two-photon detection of ClO^−^. **(D)** Working principle of AIE probe **7** for fluorescent two-photon detection of ALP and **(E)** two-photon imaging of HeLa multicellular tumor spheroids (reproduced with permission from Li H. et al., 2020; Copyright 2020 Wiley-VCH.).

As a signal gas transmitter, H_2_S plays a vital role in regulating the different functions of life systems (Hong et al., 2018; Lv L. et al., 2020; Wang J. et al., 2020; Zhang D. et al., 2020). Yoon's group developed a two-photon AIE probe **1** based on the mechanism of excited-state intramolecular proton transfer (ESIPT) for fluorescent turn-on detection of H_2_S (Figure 2A) (Chen et al., 2017). The probe **1** showed almost no fluorescence due to free motion and inhibited ESIPT effect. After reaction with H_2_S, the six-membered pyran ring of probe **1** opened and the H atom in the phenolic hydroxyl group was bonded with the N atom in the adjacent benzothiazole to form compound **2**, which showed dramatically enhanced fluorescence (about 80-fold) because of ESIPT and AIE characteristics. The detection limit was calculated to be 41 nM. The probe **1** showed high selectivity and sensitivity toward H_2_S. The authors further applied the probe **1** for two-photon fluorescence imaging of H_2_S in HeLa cells, rendering its high potential in investigating the biological and pathological roles of H_2_S in the life system.

The changes of pH and viscosity in cells will affect the reaction rate and material and energy transfer rates of cells, which have important biological value for the analysis of various physiological activities of cells (Han et al., 2020; Kim et al., 2020; Ma et al., 2020; Zhang J. et al., 2020; Zhi et al., 2020). A novel AIE-active probe **3** based on a styrylquinoline derivative with excellent two-photon performance was designed and synthesized (Figure 2B) (Dou et al., 2019). Due to the AIE effect, the probe emitted yellow emission of 550 nm in solution. Based on the response principle of N atom in quinoline to H^+^ (compound **4**), the probe **3** exhibited good sensitivity toward pH with blue emission of 470 nm and a pKa value of 3.21. It also showed a good linearity with pH in the range 3.0–1.25. In addition, due to its free motion feature, the AIE probe displayed enhanced yellow emission at 550 nm at high viscous solution (compound **3'**). The two-photon absorption cross section was very high (up to 612 GM at 640 nm). Further two-photon bioimaging of viscosity and pH in live HeLa cells was successfully carried out.

As one of the important reactive oxygen species, hypochlorite (ClO^−^) plays a very key role in modulating a set of biological processes in the life systems, such as signal transduction, inflammation, carcinogenesis and neurological diseases. Proper concentration can be highly effective for antibacterial, anti-inflammatory and pro-inflammatory properties (Hong et al., 2019; Feng A. et al., 2020; Li L. et al., 2020; Zhong et al., 2020). However, the abnormal concentration of ClO^−^ can react with various components, leading to cell damage, tissue destruction, and eventually many diseases. Ding et al. reported a two-photon AIE probe **5** for ClO^−^ visualization (Figure 2C) (Zhang et al., 2019). The probe **5** emitted almost no fluorescence, due to strong intramolecular motion. After reaction with ClO^−^, the ethylene bonded to indole quaternary salt was destructed to result in compound **6** with significant blue-shift emission of 514 nm. The probe showed fast response and a low detection limit of 13.2 nM toward ClO^−^. The two-photon absorption cross section of probe **5** after reaction with ClO^−^ was 15.3 GM at 730 nm. Additionally, the probe showed a good two-photon imaging performance for turn-on detection of ClO^−^ in HeLa cells.

Alkaline phosphatase (ALP) is considered to be a key biomarker related to signal transduction and tumor metabolism. The overexpression of ALP is closely related to the occurrence, development and deterioration of tumor and can be used as one of the important clinical indexes (He et al., 2020c; Khatun et al., 2020; Xu J. et al., 2020). Yoon and Peng et al. developed a new two-photon probe **7** based on typical AIE building block quinoline-malonitrile (QM) for ALP monitoring and surgical tumor excision (Figure 2D) (Li H. et al., 2020). The amphiphilic water-soluble probe **7** self-assembled to form nanoaggregate in aqueous solution. Upon addition with ALP, the fluorescence of self-assembled compound **8** was boosted, and the detection limit was calculated to be 0.15 mU/mL. The nanoprobe **7** could selectively light-up HeLa cells with overexpression of ALP, and was applied to monitor the down-regulation and up-regulation of ALP activity. Long-term high-fidelity cell imaging of up to 13 h was achieved. The authors used this probe for two-photon imaging of HeLa multicellular tumor spheroids with high penetration depth. In addition, the probe was also successfully used to distinguish tumor tissues from normal tissues in BABL/c mice bearing HepG-2 and HeLa xenografts and further to guide tumor resection.

### *In vitro* Cell Imaging

Cells are one of the basic components of organisms. Each cell is composed of various organelles such as plasma membrane, mitochondria, lysosome, golgi apparatus, endoplasmic reticulum, nucleus, centrosome, microtubule, and microfilament. These structures are very important to the precise operation of cells and even the whole body, and diverse biochemical processes and physiological pathways that occur in life have more profound significance. Thus, the development of organelle-specific biological probes makes it possible to study specific target organelles, which can reveal the location and morphology of organelles and monitor the changes of important physiological activities or organelle dysfunction for researchers.

**Figure 3:**
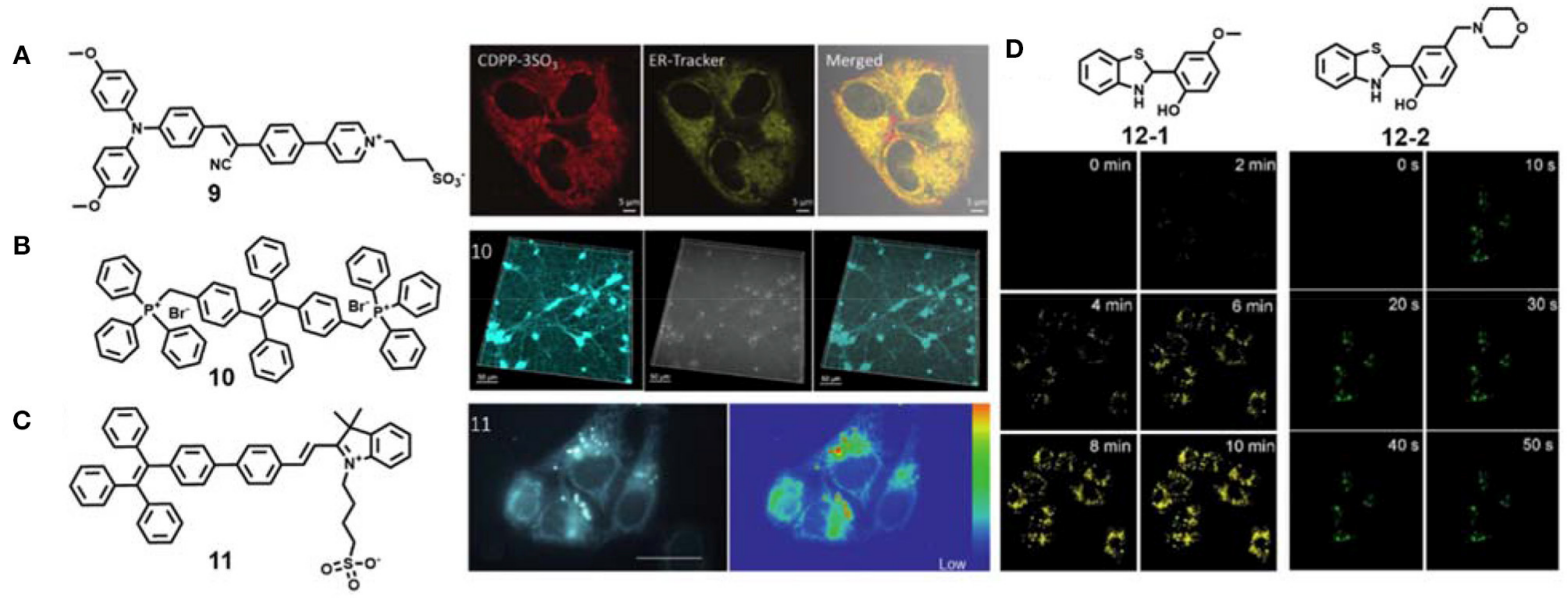
**(A)** Chemical structure of AIE material **9** and two-photon endoplasmic reticulum imaging in live cells (reproduced with permission from Alam et al., 2020; Copyright 2020 Wiley-VCH). **(B)** Chemical structure of AIE material **10** and two-photon mitochondria imaging in live cells (reproduced with permission from Qian et al., 2015; This figure is extracted from an open access journal with thanks; Copyright 2015 The Optical Society). **(C)** Chemical structure of AIE material **11** and two-photon mitochondria and lipid droplet lifetime imaging in live cells (reproduced with permission from Chen et al., 2015; Copyright 2015 Wiley-VCH). **(D)** Chemical structures of AIE material **12-1** and **12-2** and two-photon photoactivated imaging of lipid droplets and lysosomes, respectively, in live cells (reproduced with permission from Li et al., 2018; This figure is extracted from an open access journal with thanks; Copyright 2018 The Royal Society of Chemistry).

As an important organelle in cells, endoplasmic reticulum plays a key role in cell metabolism, protein synthesis and transmission of intermediates and signal molecules (Zhang M.-M. et al., 2020; Zhao et al., 2020). Tang's group reported an AIE material **9** based on cyanostillbene skeleton by simple functionalization (Figure 3A) (Alam et al., 2020). Interestingly, the zwitterionic donor-receptor-type AIE material **9** with positive-charged pyridinium and negative-charged sulfonic group can specifically target the endoplasmic reticulum in live cells. This AIE material **9** showed maximum emission wavelength under 620 nm, two-photon absorption cross section of about 120 GM, and solid-state quantum efficiency of as high as 39.3%. The imaging pattern of AIE material **9** was well overlapped with that of ER-Tracker Red with a high Pearson's correlation value of 0.85. Further two-photon bioimaging of endoplasmic reticulum under 820 nm excitation was achieved.

Mitochondria are the “power factories” in cells, and their defects are closely related to aging, cancer and neurodegenerative diseases, including Alzheimer's disease, Huntington's disease and Parkinson's disease (Baines, 2010). Generally, positively charged fluorescent dyes can selectively locate in mitochondria *via* electrostatic interaction (Zhang et al., 2015; Jean et al., 2016; Liu et al., 2016). The photostable AIE material **10** was also confirmed to stain mitochondria in live HeLa cells (Figure 3B) (Qian et al., 2015). To expand its further application, Qian et al. applied these materials for two-photon imaging in primary neurons and mouse brain microglia. Because of high photostability of compound **10** in an aggregate state, they achieved long-term neuroimaging. Besides traditional fluorescence imaging, fluorescence lifetime imaging was also performed for mitochondria visualization. Chen et al. used the AIE material **11** for two-photon lifetime imaging of dual organelles: mitochondria and lipid droplets (Figure 3C) (Chen et al., 2015). The imaging data revealed that mitochondria incubated with compound **11** show longer lifetimes than lipid droplets. The authors further mapped the subcellular viscosity.

Photoactivatable fluorescent probes are preferable tools for organelle study with remarkably increased spatiotemporal resolution (Fay et al., 2020; Zou et al., 2020). Tang and Gao et al. developed two photoactivated AIE probes **12-1** and **12-2** for specific organelle imaging (Figure 3D) (Li et al., 2018). These AIE probes were *in situ* generated from easily available disulfide and thiol substrates through tandem S–S bond reduction and intramolecular cyclization reaction. Their subcellular organelle targeting ability could be regulated by the functionalized groups on the skeleton core. The probes **12-1** and **12-2** could selectively stain lipid droplets and lysosomes, respectively, and high-resolution photoactivated imaging under one-photon and two-photon irradiation were also achieved. It should be noted that the fluorescence of the probes **12-1** and **12-2** can be activated faster under two-photon excitation than one-photon excitation.

### *Ex vivo* Tissue Imaging

*In situ* and direct imaging of organelles in intact tissue can provide more intrinsic and accurate information than cells *in vitro*. On the one hand, the biological environments of the two are different. The cells in the live tissues are in the extracellular matrix, while the cells *in vitro* are in the artificial culture medium. On the other hand, the cells *in vitro* are generally immortalized cells that can be infinitely added, and their original differentiation function has been destroyed. In a word, the process of *ex vivo* tissue imaging is more complex and difficult than that of *in vitro* cell imaging, and the requirements of probes are more stringent.

**Figure 4:**
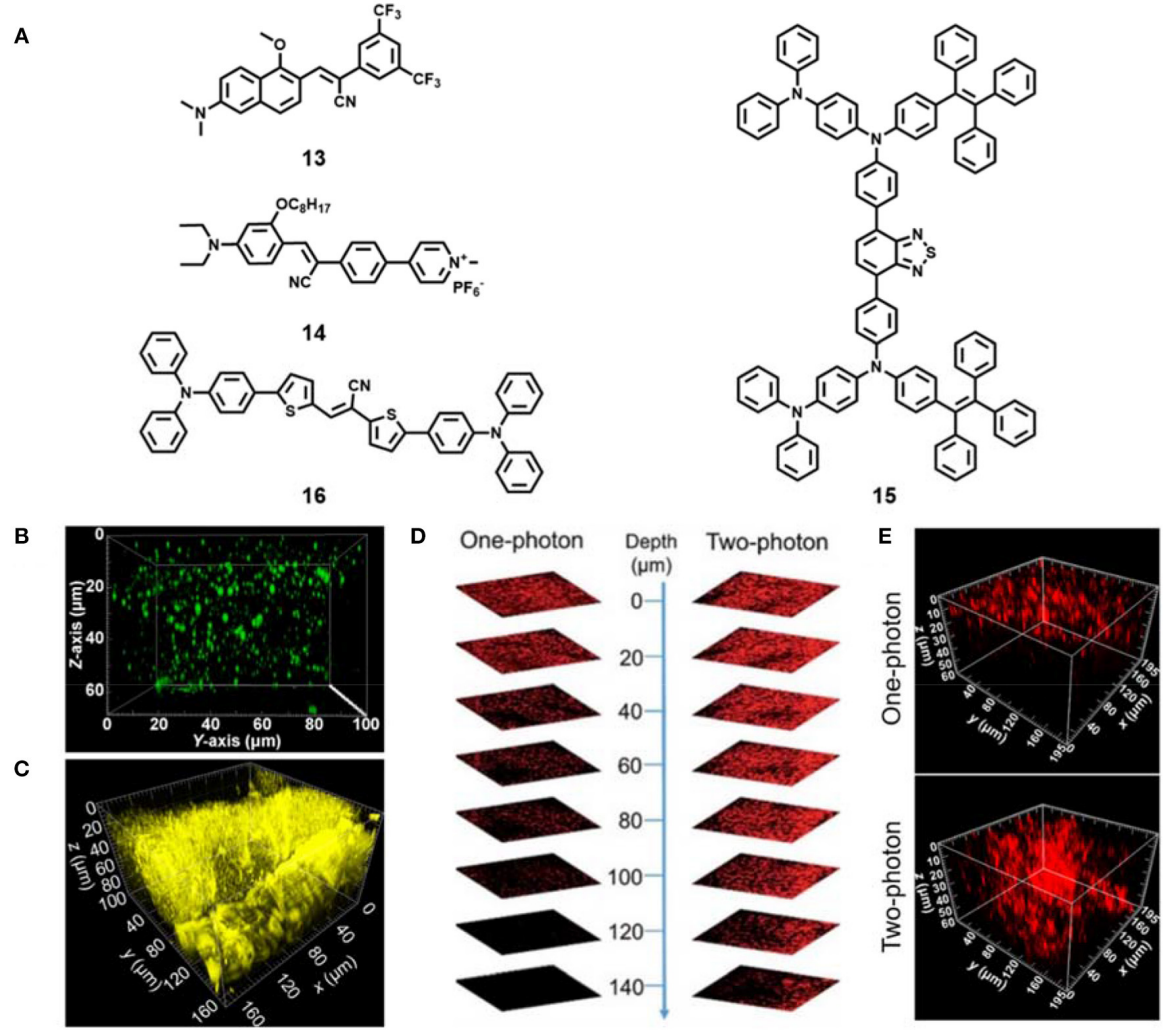
**(A)** Chemical structures of AIE materials **13-16** for two-photon tissue imaging. **(B)** Reconstructed 3D two-photon fluorescence imaging of lipid droplets in live mouse liver tissues incubated with AIE material **13** (reproduced with permission from Niu et al., 2018; Copyright 2018 American Chemical Society). **(C)** Reconstructed 3D two-photon fluorescence imaging of live mice muscle tissues incubated with AIE material **14** (reproduced with permission from Niu et al., 2019a; Copyright 2019 Elsevier). **(D)** One-photon and two-photon fluorescent images of mouse liver by intravenous injection with compound **15** based AIE dots (reproduced with permission from Qin et al., 2018; This figure is extracted from an open access journal with thanks; Copyright 2018 The Royal Society of Chemistry). **(E)** Reconstructed 3D One-photon and two-photon fluorescent images of mouse tumor by intratumor injection with compound **16** based AIE NPs (reproduced with permission from Niu et al., 2019b; Copyright 2019 American Chemical Society).

Recent studies showed that hydrophobic dyes with high logP (n-octanol/water partition coefficient) value are inclined to locate in lipid droplets (Zhao et al., 2019; Wang L. et al., 2020; Ye et al., 2020; Zhang F. et al., 2020; Zhang X. et al., 2020). A few fluorescent dyes have been applied for two-photon lipid droplet imaging in live cells and fixed tissues (Collot et al., 2018; Fam et al., 2018). However, fluorescent probes for two-photon imaging of lipid droplets in live tissues are rarely reported. Tang and Yu et al. synthesized a group of two-photon AIE fluorescence probes capable of selective staining lipid droplets in live cells and live tissues (Niu et al., 2018). These AIE probes exhibited excellent photophysical properties: large Stokes shift (>100 nm), high solid-state fluorescence quantum yield (30%), good two-photon absorption cross section (45–100 GM at 860 nm), high biocompatibility and excellent photostability. Rapid and specific staining lipid droplets in live cells at an ultralow concentration (50 nM) was achieved. Such concentration is the lowest value for lipid droplet staining in live cells reported so far. The authors used probe **13** (Figure 4A) as an example to successfully achieve two-photon specific imaging of lipid droplets in live mouse liver tissues at a depth of about 70 μm (Figure 4B).

Later, Tang's team developed a family of far-red and NIR AIE materials based on a carbazole-bridged push–pull framework (Zheng et al., 2018). Among these AIE materials, the compound DCMa could two-photon image lipid droplets at the depth of 129 μm in live mouse liver tissues, and the compound DCPy showed two-photon deep-tissue imaging of mitochondria in live mouse muscle tissues (77 μm). Subsequently, the positively charged AIE material **14** (Figure 4A) was also reported in their group (Niu et al., 2019a). The compound **14** showed large Stokes shift (>180 nm), high fluorescence quantum yield (12.8–13.7%), and excellent photostability under both one- and two-photon continuous irradiation. Moreover, the probe achieved the *ex vivo* two-photon depth tissue imaging of rat skeletal muscle mitochondria at the penetration depth of up to 100 μm (Figure 4C).

Besides small AIE dyes, AIE nanomaterials by a simple nanoprecipitation method were also fabricated for *ex vivo* two-photon tissue imaging (Liu et al., 2019). Recently, Tang's group prepared red emissive AIE dots based on an efficient solid-state red-emissive AIE compound **15** (Figure 4A) with a high fluorescence quantum yield of 34.1% (Qin et al., 2018). The AIE dots exhibited a large two-photon absorption cross section value of 310 GM at 900 nm. The deep-tissue imaging performance of the AIE dots in mouse liver excited by a two-photon pulse laser (>200 μm) was obviously better than that of one-photon imaging (<100 μm) (Figure 4D). They also demonstrated their two-photon deep-tissue imaging of the blood vessels in mouse ears and brain. Later, they unexpectedly developed a red-emissive organic AIE material **16** (Figure 4A) with excellent fluorescence quantum yield (37.6%), large two-photon absorption cross section (508 GM) and high biocompatibility (Niu et al., 2019b). The fabricated AIE nanoparticles (NPs) based on compound **16** could specifically stain lysosomes in live cells. In addition, high-resolution two-photon deep tissue imaging of this AIE NPs in tumor tissue under 880 nm excitation was obtained, and the two-photon imaging performance was better than one-photon excitation (Figure 4E). Further use of this NPs for long-term imaging of tumors in mice was achieved. The AIE NPs show great potential in two-photon deep tissue bioimaging and long-term dynamic tracking of tumor metastasis.

### *In vivo* Vasculatures Imaging

Blood vessels play an essential role in the growth and metastasis of solid tumors, and can transport nutrients and oxygen in the tumor microenvironment (Dewhirst and Secomb, 2017; Li et al., 2021). Tumor blood vessels are usually characterized by structural and functional abnormalities, vascular leakage, dilatation, bending of blood vessels, increasing non-uniformity of tumor blood flow, and so on (Tozer et al., 2005). Cerebrovascular abnormalities are related to cerebrovascular diseases such as stroke, vascular malformations, aneurysms, and transient ischemic attacks (Chen C.-J. et al., 2018; Lin et al., 2018). Therefore, the utilization of an *in vivo* two-photon fluorescence microscope with cell resolution and promising sensitivity can not only effectively identify and monitor the vascular structure, morphology, and normalization process of cerebrovascular systems and tumors, but also provide valuable information for the diagnosis and treatment of the disease. Moreover, this method reduces the light absorption and scattering of biological tissue, and achieves better light transmittance and deeper imaging depth.

**Figure 5:**
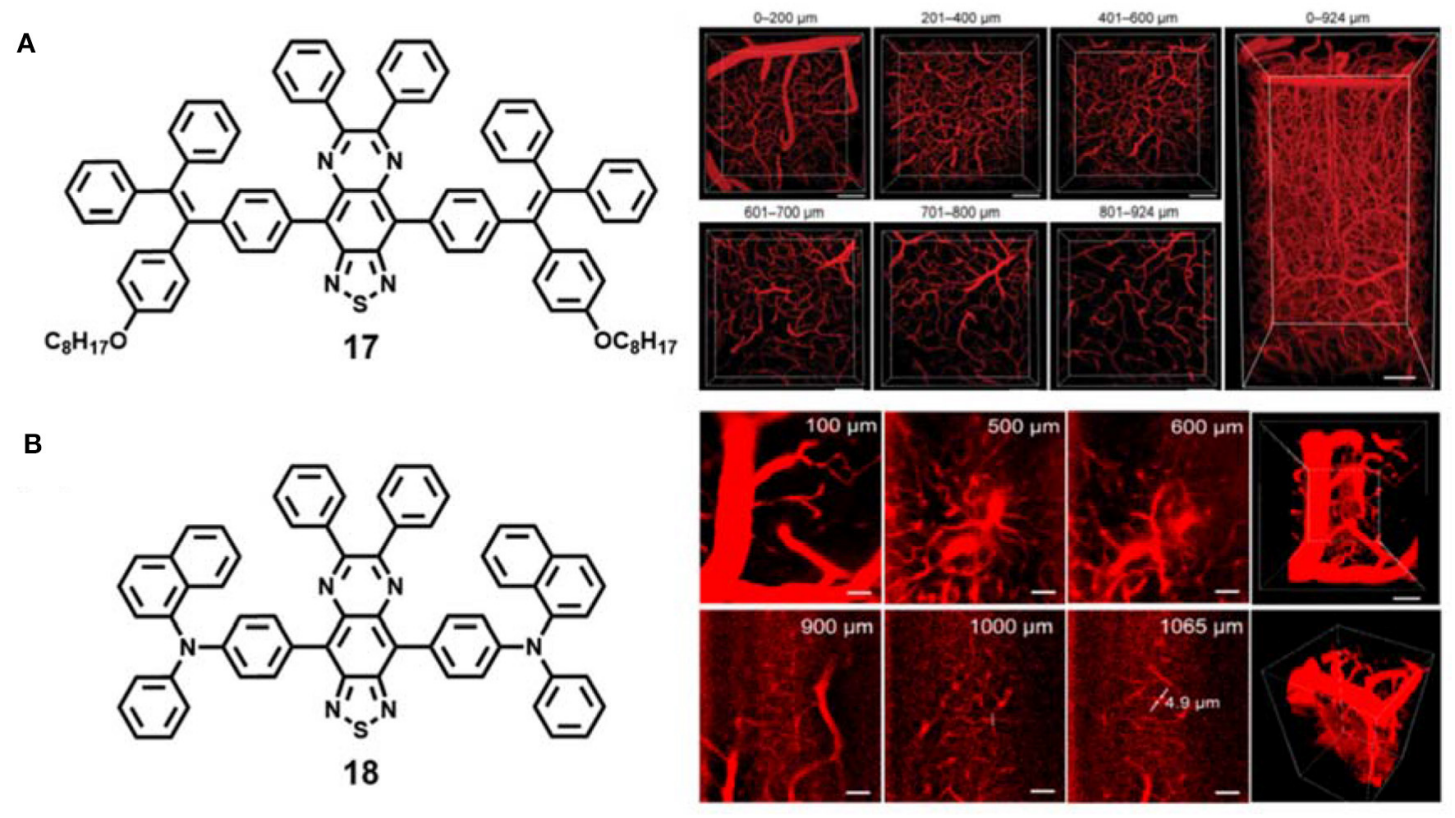
**(A)** Chemical structures of AIE material **17** and 3D two-photon intravital imaging of mouse brain vasculature network at different penetration depths (reproduced with permission from Wang et al., 2019b; Copyright 2019 Wiley-VCH). **(B)** Chemical structures of AIE material **18** and 3D two-photon imaging of mouse brain blood vessels at different penetration depths (reproduced with permission from Qi et al., 2018; Copyright 2018 American Chemical Society).

Liu and co-authors developed efficient near-infrared AIE dots based on the compound **17** (Figure 5A) (Wang et al., 2019b). The fabricated AIE dots showed excellent properties: high quantum yield (19 ± 1%) and ultralarge two-photon absorption cross section (7.63 ×10^4^ GM at 1,200 nm). Under the two-photon NIR-II excitation (1,200 nm), the mouse brain vasculatures labeled by the AIE dots could be clearly imaged at an ultradeep depth of 924 μm (Figure 5A). They successfully used AIE dots to distinguish the tumor vessels from normal vessels, and realized the non-invasive real-time imaging of the deep tumor vascular network with large penetration depth (670 μm) and high signal-to-background ratio (about 120) under the two-photon NIR-II excitation. This work opens up a new avenue for effective near-infrared materials excited by two-photon NIR-II light *in vivo* tumor imaging.

Tang and Qian et al. fabricated other AIE dots based on red-shifted NIR emissive AIE compound **18** with a maximum emission wavelength of 810 nm for ultradeep intravital two-photon fluorescence bioimaging (Figure 5B) (Qi et al., 2018). The water-soluble AIE dots exhibited superior large two-photon absorption cross section (1.22 ×10^3^ GM at 1,300 nm). Under two-photon 1,300 nm NIR-II light irradiation, 3D blood vessels outside white matter (>840 μm) and even in the hippocampus (>960 μm) were successfully constructed at ultrahigh spatial resolution (<3.5 μm), and 5 μm vessels in the mouse brain were clearly visible at the depth of 1,065 μm (Figure 5B), which was one of the deepest penetration depths and the best spatial resolution reported so far.

## Conclusion and Outlook

AIE materials have become a preferential choice for two-photon fluorescence bioimaging because of their strong fluorescence and excellent photostability in high concentration or aggregation states. This review mainly summarizes the recent advances of AIE-active materials for two-photon bioimaging with some representative examples in four areas: fluorescence detection, *in vitro* cell imaging, *ex vivo* tissue imaging, and *in vivo* vasculature imaging. Though some interesting progress has been achieved, there are some unsolved problems and challenges which still need to be overcome.

New recognition units need to be rationally incorporated into the skeleton of AIE-active two-photon materials to extend the analyte detection ranges, such as DNA, protein, enzyme, chiral molecules, and so on. However, for AIE probes, the detection limit is a critical parameter to be carefully considered. In addition, subcellular organelle targeted AIE-active two-photon materials should be switched to some other important organelles like golgi apparatus, nucleus, and nucleolus, which could be possibly achieved by conjugation with specific organelle-targeting peptides. It should be noted that the specificity of these organelle targeted AIE-active materials could be altered a lot in neuron cells. In some point, the development of such materials for specific organelle targeting in neuron cells is challenging. The majority of AIE materials exhibit small to moderate two-photon absorption cross section, so enhancing the two-photon absorption cross section is still an important issue. Generally, extending the π-conjugation length of AIE skeletons and introducing strong electron donor and acceptor groups to enhance the intramolecular charge transfer effects are two widely adopted strategies to improve the two-photon absorption as well as to enable the red shift of absorption and emission. However, these strategies can result in high hydrophobicity of AIE materials. The balance between hydrophobicity and penetrability to live samples needs careful consideration. On the other hand, the twisted intramolecular charge transfer (TICT) effect should be also considered, as the TICT effect is detrimental for AIE materials especially NIR emissive ones. It should be also added that the fabrication of composite two-photon AIE-active materials with other materials like inorganic quantum dots is an effective method to render the composite materials with multifunctionalities, such as multimode imaging and activity-based cancer diagnosis. Furthermore, traditional chemotherapy and modern phototherapy (photodynamic and photothermal therapy) based on drugs and photosensitizers, respectively, have been demonstrated to show satisfying therapeutic effects. Combined with two-photon fluorescence imaging of AIE-active materials, such imaging-guided therapy could emerge as an effective modality for precise spatiotemporal control of cancer therapy. Therefore, there is still much room to improve and develop many fascinating AIE-active materials for diverse two-photon biomedical imaging. We hope this review can shed new light on future two-photon bioimaging application of AIE-active materials.

## Author Contributions

All authors listed have made a substantial, direct and intellectual contribution to the writing process of this article, and approved the manuscript.

## Conflict of Interest

The authors declare that the research was conducted in the absence of any commercial or financial relationships that could be construed as a potential conflict of interest.
